# Erectile dysfunction and metabolic syndrome components in obese men with psoriasis: response to a 12-week randomized controlled lifestyle modification program (exercise with diet restriction)

**DOI:** 10.1007/s11845-023-03412-8

**Published:** 2023-05-31

**Authors:** Ali Mohamed Ali Ismail, Dina E. Hamed

**Affiliations:** 1 Department of Physical Therapy for Cardiovascular/Respiratory Disorders and Geriatrics, Faculty of Physical Therapy, Cairo University, Giza, Egypt; 2 Department of Dermatology, Venereology, and Andrology, Faculty of Medicine, Zagazig University, Zagazig, Egypt

**Keywords:** Erectile dysfunction, Exercise, Low calorie diet, Metabolic syndrome, Psoriasis

## Abstract

**Background:**

Erectile dysfunction (ED) and metabolic syndrome (MeTS) are highly prevalent in chronic plaque psoriasis (CPP).

**Objective:**

The aim of this lifestyle modification study is to explore the response of MeTS components and ED to a 12-week lifestyle modification program (low-calorie diet and moderate-intensity treadmill walking) in 60 obese men with CPP, mild and moderate ED, and MeTS.

**The design, settings, participants, and intervention:**

In this lifestyle modification randomized study, a university-based hospital recruitment of 60 obese men with CPP, mild and moderate ED, and MeTS was randomly performed. Men were randomly assigned to the lifestyle modification group (*n* = 30, received low-calorie diet and moderate-intensity treadmill walking programs) or waitlist group (*n* = 30). The following outcomes were assessed as follows: body mass index, psoriasis severity (assessed via psoriasis area and severity index), ED (assessed by the five-item internal index of erectile function), and components of MeTS (waist circumference, blood pressure, serum high-density lipoprotein, serum triglycerides, and serum fasting blood glucose).

**Results:**

Trends of significant improvements in all outcomes were documented in favor of the lifestyle modification group. All outcomes of the waitlist group did not show the same reported significant improvements of the lifestyle modification group.

**Conclusion:**

A 12-week lifestyle modification program as a tool for weight loss in obese men with CPP is a good therapeutic method to improve psoriasis severity and psoriasis-associated ED and MeTS.

## Introduction

The autoimmune-mediated inflammatory skin disease in 2 to 3% of the general adult population is chronic plaque psoriasis (CPP). In men, CPP is strongly associated with erectile dysfunction [1] (ED, inability to initiate/maintain penile erection sufficient for good/satisfactory sexual intercourse) [2, 3] and metabolic syndrome [4] (MetS, a metabolic/vascular cluster of abdominal/visceral obesity, hypertension, atherosclerosis-induced dyslipidemia, and insulin resistance and/or type 2 diabetes) [5].

The prevalence of CPP-associated MetS ranges from 20 to 50%. Compared with non-psoriatic age-matched control subjects, the risk of having MetS is at least double in psoriatic sufferers [4]. Also, while ED is found in 58% of CPP men, it is found in a lower percentage, 49%, in the non-psoriatic age-matched control men [6].

The mechanism links between ED, MeTS, and CPP might be contributed to the chronic systemic inflammatory nature of CPP. This chronic inflammation is a predisposing factor to the development of endothelial dysfunction in CPP. Endothelial dysfunction—chronic vascular inflammation combined with nitric oxide dysfunction and vascular insufficiency—is the same pathogenic mechanism that explains the highly prevalent ED and MeTS in CPP [7].

Recently, published psoriatic reports confirm that patients’ correct lifestyle (proper diet with increased physical activity levels) may positively affect the clinical signs/symptoms, severity/progression, outcomes of CPP [8], and CPP-associated comorbidities such as ED [9] and cardiovascular diseases including MeTS [8].

Low-calorie diets (LCD) seem to result in an improved body mass index (BMI), CPP activity, pharmacotherapies’ efficacy, and CPP-associated cardiovascular comorbidities such as MeTS, especially in sufferers who are overweight or obese [10]. Increased physical activity—as a fundamental/complementary approach in LCD-induced weight loss programs—may lead to a maximization of the above-mentioned improvements after following LCD in obese CPP patients [8]. Also, considering ED is a predictor or risk factor for cardiovascular diseases including MeTS [11], exercise-induced improvement of metabolic profile (blood glucose and lipids), and blood pressure in cardiovascular disease risk patients [12] could be utilized in sufferers with ED and CPP [9].

Since there are no published studies to clarify the effect of lifestyle changes (exercise and diet intervention) on ED in psoriasis patients [9], this lifestyle modification study aims to explore the response of MeTS components and ED to a 12-week lifestyle-modification program in obese men with ED, CPP, and MeTS.

## Materials and methods

### Settings

The men diagnosed with CPP, ED, and MeTS were randomly recruited from Cairo University Hospitals. Recruitment of the subjects was conducted during the period between 16th October 2022 and 16th April 2023.

### Men’s inclusion criteria

With ages ranging from 32 to 49 years old (because these age ranges were the available age ranges of CPP men who visited the recruitment settings during the recruitment period), this lifestyle modification trial included 60 mild-to-severe CPP married men with ED. The erectile function in psoriatic men was evaluated using the Arabic version of the five-item international index of erectile function questionnaire (IIEFQ-5). Psoriatic men with IIEFQ-5 scores ≥ 8 to < 21 (i.e., mild to moderate ED) were included. Men’s BMI ranged from 30 to 34.9 kg/m^2^.

The psoriatic men who showed at least three MeTS components were included in this lifestyle modification study. The components were men’s waist circumference (WC) ≥ 102 cm, blood pressure (BP) ≥ 130/85 mmHg or regular administration of pharmacological therapies to high BP, the value of serum high-density lipoprotein (SHDL) < 40 mg/dl, value of serum triglycerides (STG) ≥ 150 mg/dl, and serum fasting blood glucose (SFBG) level—tested after 6-h fasting— ≥ 110 mg/dl [4]. This lifestyle modification study in obese men with CPP, MeTS, and ED was registered on clinicaltrial.gov (NCT05632042).

All ethical issues concerned with Helsinki recommendations, institutional approval, and men’s consent were followed by the two authors. This single-blinded lifestyle modification approach in CPP men with MeTS and ED received local institutional approval (P.T.REC/012/004117).

### Men’s exclusion criteria

Men who received phototherapeutic interventions, weight loss prescriptions (diet, exercise, or drugs), biologic therapies, and pharmacological inhibitors of phosphodiesterase-type 5 (Is-PDET5) within the last 12 weeks were excluded. CPP men with autoimmune or neurological disorders, hypogonadism (low testosterone levels), hepato-renal or respiratory disorders, penile implants, cardiopulmonary or vascular disorders, structural deformations of the penile shaft, lower limb arthritic disorders, prostatic disorders, mental disorders, drinking or smoking habits, or tumors were excluded from this lifestyle modification study.

### Randomization

**Fig. 1:**
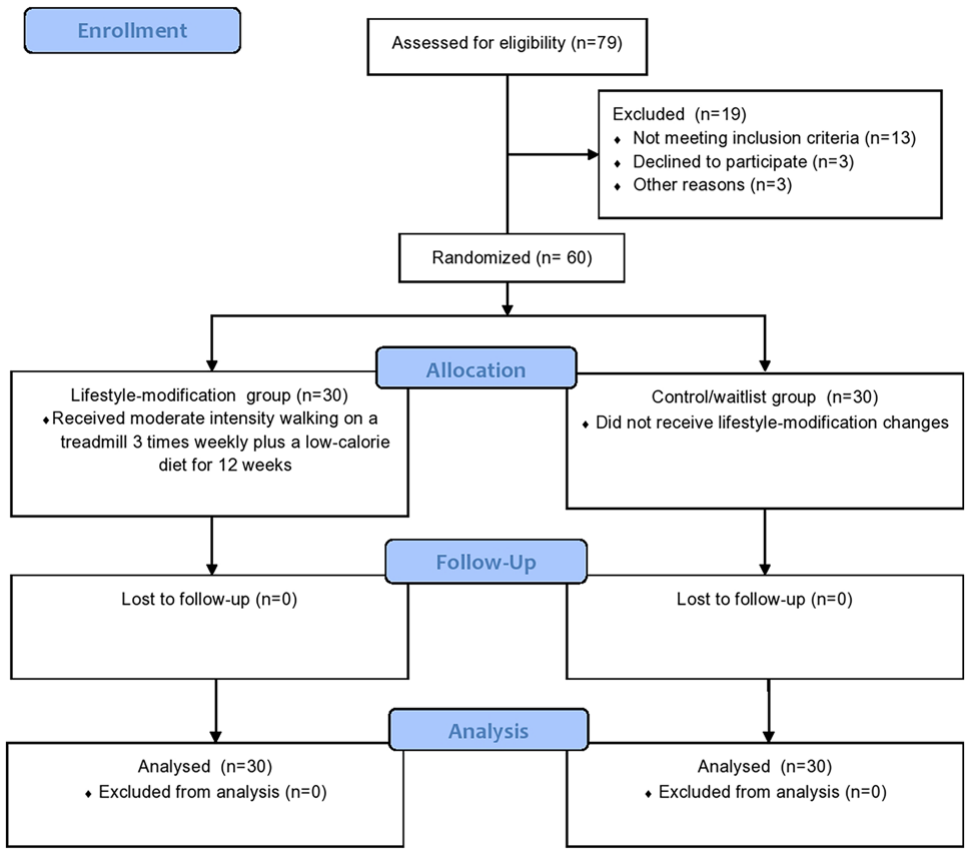
Consort flow chart of the lifestyle modification approach in obese men with psoriasis, erectile dysfunction, and metabolic syndrome

The consented psoriatic men with MeTS and ED were divided by the simple randomization technique, a closed envelope technique conducted by a physiotherapy assistant who was unaware of the lifestyle changes that would be executed, into two 30-men groups. Men in the first group (lifestyle modification group, LMG) were treated with a low-calorie diet (LCD) and supervised moderate-intensity exercise program for 12 weeks, while men in the second group (control group, CG) were ordered not to change their lifestyle habits (Fig. 1).

### Lifestyle modification program

Harris–Benedict equation—[(10 × weight of men in kilograms) + (6.25 × height of men in centimeters) − (5 × age of men in years) + 5]—was utilized to calculate participants’ basal metabolic rate (BMR) before involving them in the low-calorie-diet protocol. To start the low-calorie diet, the value of BMR was subtracted from 500 kilocalories per day [13]. Besides fruits (such as peach, apple, pear, strawberries, plum, guava, and pineapple) and vegetables, macronutrients (20–30% fats, 10–15% proteins, and 55–65% carbohydrates) formed the 3-meal diet plan daily [13, 14]. To monitor the adherence of psoriatic men with MeTS and ED to the introduced energy restriction program, the low-calorie diet was reviewed weekly through a face-to-face interview.

The LMG received a supervised 40-min on-treadmill walking program. Day after day (3 times weekly), the session was constituted of 3 stages in all obese men with CPP, ED, and MeTS. Warming-up and cooling-down walking stages conducted at 40–50% of men’s maximal heart rate, 5 min for each, were done before and after the main exercise stage (a 30-min walking on the treadmill at 60–70% of men’s maximal heart), respectively.

### Main outcome (Arabic version of IIEFQ-5)

A hard copy of IIEFQ-5 was given to every psoriatic man to fill it before and after the 12-lifestyle-modification program.

### Secondary outcomes

Besides BMI, MeTS components of all psoriatic men were tested. The components included men’s BP (systolic and diastolic pressures were assessed with a manual sphygmomanometer), WC (evaluated via in elastic tape at men’s umbilicus level), SFBG (assessed with a glucose blood meter after overnight fasting), and overnight fasting STG and SHDL. Also, the psoriasis area and severity index (PASI) was evaluated in all participating men.

The second author used the validated PASI to assess psoriasis severity before and after this 12-week lifestyle modification study. With a score ranging from 0 to 72, PASI assessed men’s erythema/scaliness levels and the density of CPP in men’s head, neck, upper/low limbs, and trunk. If there was a decrease in PASI scores after ending the lifestyle modification protocol, it was considered an improvement in CPP severity.

### Blinding

Assessors of the primary outcome (IIEFQ-5) or secondary outcomes (BMI, MeTS components, and PASI) were not informed of the details of the 12-week lifestyle modification program introduced to the examined 60 CPP men with MeTS and ED.

#### G*power sample size

Sixteen obese men with CPP, MeTS, and ED were the pilot test that was used to extract the needed sample size calculation at a power of 80%. The effect size of IIEFQ-5 (*d* = 0.77) detected the need for 54 obese men with CPP, MeTS, and ED. The two authors tried to avoid a 10% estimated dropout of patients during the application of the 12-week lifestyle modification program by adding extra 6 obese men with CPP, MeTS, and ED.

#### Statistical analysis (SPSS 18)

The Smirnov test—used to examine the distribution of primary outcome (IIEFQ-5) or secondary outcomes (BMI, MeTS components, and PASI)—confirmed the normal distribution of CPP men’s primary and secondary outcomes, so ANOVA test (repeated measure) was used to assess the statistical changes within and between groups, respectively in this 12-week lifestyle modification study.

## Results

Before conducting the 12-week lifestyle modification program, using the ANOVA test, comparison of demographic data (Table 1, only the non-significant difference of men’s between-group ages were confirmed by unpaired test), the primary outcome (IIEFQ-5), or secondary outcomes (systolic/diastolic BP, SFBG, SHDL, STG, PASI, and WC) did not show a significant difference between LMG and CG (Table 2).

**Table 1:** Basic data (mean ± standard deviation) of metabolic syndrome men with psoriasis and erectile dysfunction

**Men’s basic data**	**Lifestyle modification group**	**Control group**	***p*** **value**
**Men’s age** (year)	44.03 ± 3.59	44.80 ± 3.17	0.382
**Men’s body mass index** (kg/m^2^)	31.85 ± 1.39	32.39 ± 1.38	0.136
**Men’s waist circumference (cm)**	107.03 ± 14	109.46 ± 12.21	0.476

*p* value is > 0.05 so a non-significant between-group basic data is confirmed

**Table 2:** Mean ± SD outcomes of metabolic syndrome men with psoriasis and erectile dysfunction

**Men’s outcomes**	**Lifestyle modification group**	**Control group**	***p*** **value (among-groups)**
**Men’s pre body mass index (kg/m**^**2**^**)**	31.85 ± 1.39	32.39 ± 1.38	0.136
**Men’s post body mass index (kg/m**^**2**^**)**	29.93 ± 1.57	32.49 ± 1.58	0.0001*
*p* value “within-men groups”	<0.001*	0.573	
**Men’s pre waist circumference (cm)**	107.03 ± 14	109.46 ± 12.21	0.476
**Men’s post waist circumference (cm)**	100.53 ± 14.17	110.10 ± 11.57	0.006*
*p* value “within-men groups”	<0.001*	0.322	
**Men’s pre fasting blood glucose (mg/dl)**	109.36 ± 29.34	118.30 ± 34.62	0.285
**Men’s post fasting blood glucose (mg/dl)**	100.50 ± 27.14	118.60 ± 32.80	0.023*
*p* value “within-men groups”	<0.001*	0.778	
**Men’s pre triglycerides (mg/dl)**	157.33 ± 98.26	166.30 ± 92.18	0.717
**Men’s post triglycerides (mg/dl)**	120.70 ± 76.97	167.93 ± 97.47	0.042*
*p* value “within-men groups”	<0.001*	0.761	
**Men’s pre high-density lipoprotein (mg/dl)**	40.43 ± 10.77	38.60 ± 11.09	0.519
**Men’s post high-density lipoprotein (mg/dl)**	44.83 ± 12.26	38.03 ± 10.97	0.027*
*p* value “within-men groups”	<0.001*	0.435	
**Men’s pre systolic pressure (mmHg)**	124.96 ± 17.29	128.63 ± 16.74	0.408
**Men’s post systolic pressure (mmHg)**	120.20 ± 15.85	129.33 ± 17.34	0.038*
*p* value “within-men groups”	0.001*	0.594	
**Men’s pre diastolic pressure (mmHg)**	83.26 ± 9.86	84.46 ± 10.40	0.648
**Men’s post diastolic pressure (mmHg)**	79.70 ± 7.91	85.13 ± 12.37	0.047*
*p* value “within-men groups”	<0.001*	0.474	
**Men’s pre psoriasis area and severity index**	7.56 ± 4.63	9.90 ± 5.10	0.069
**Men’s post psoriasis area and severity index**	5.56 ± 4.46	10.06 ± 4.51	< 0.001*
*p* value “within-men groups”	<0.001*	0.594	
**Men’s pre IIEFQ-5**	16.13 ± 3.70	17.23 ± 3.66	0.252
**Men’s post IIEFQ-5**	19.06 ± 4.40	16.96 ± 3.28	0.041*
*p* value “within-men groups”	<0.001*	0.420	

*IIEFQ-5* five-item international index of erectile function questionnaire

*p value is < 0.05 so the measured outcomes in psoriatic men with erectile dysfunction and metabolic syndrome is significant

Using the ANOVA test, pre-to-post comparison of data within LMG showed a significant improvement in the primary outcome (IIEFQ-5) or secondary outcomes (systolic/diastolic BP, SFBG, SHDL, STG, PASI, and WC). Pre-to-post comparison of primary or secondary outcomes within CG did not show a significant improvement in all outcomes (Table 2).

After conducting the 12-week lifestyle-modification program, using the ANOVA test, between-group post-comparison of the primary outcome (IIEFQ-5) or secondary outcomes (systolic/diastolic BP, SFBG, SHDL, STG, PASI, and WC) shows a significant difference in favor of LMG (Table 2).

## Discussion

This is the first lifestyle modification study that reported an improvement in ED, MeTS components, and PASI after a 12-week randomized-controlled program of exercise and diet restriction in men with CPP, ED, and MeTS.

The exercise-induced decrease in psoriatic severity may be in part due to a decrease in obesity-associated high mass of adipose tissue. Decreased mass of adipose tissue is associated with a decreased release of inflammatory cytokines involved in the pathogenesis of psoriasis. Decreased production of cytokines not only restricts the causative role in the induction of CPP [15], but also improves the low-grade systemic inflammation [16] which is the common factor in the occurrence of CPP-associated comorbidities such as cardiovascular diseases (CVD), MeTS [15], and ED [9].

The suggested mechanisms that explain the improvement in MeTS components in this CPP study reduction may be reported as follows: reduction in overactivity of the sympathetic nervous system; improvement in elastic and endothelial properties/functions of vessels; enhancement functions of micro- and macrocirculation; increased production of vasodilating substances such as nitric oxide (NO), reduced production of plasma vasoconstrictors/catecholamines [17]; improved insulin resistance (may be due to repeated contraction of large muscles of lower limbs that increase the transportation, phosphorylation, and oxidization of glucose molecules during walking) [5]; and increased activity of lipolytic enzymes that catabolize STG [18, 19].

Supporting us, treatment with a low-calorie diet (a reduced food intake reaching 800–1000 kcal/day for 2 months followed by a reduced food intake reaching 1200 kcal/day for 2 months) in overweight/obese patients with CPP showed a trend in favor of clinically important improvements in BMI, WC, SFBG, PASI [16], STG, cholesterol, diastolic BP, glycated hemoglobin, and tissue plasminogen activator inhibitor [20]. Also, the median improvement in PASI of obese and overweight patients with psoriasis who followed a protocol of a 20-week low-calorie diet and exercise was greater than patients who did not follow the same protocol [21]. In overweight/obese patients with CPP, the aggressive first-line weight-loss program (very-low-calorie ketogenic diet followed by Mediterranean diet) that was followed by the patients for 10 weeks without prescribing psoriatic medications produced a significant reduction in PASI [22].

The 24-week low-calorie diet produced significant changes in BMI, WC, STG, and SHDL, but SFBG did not show significant changes despite the decrease in its level due to the small number (*n* = 10) of obese patients with CPP [23].

In obese patients with CPP, despite non-significant improvements in SHDL and STG, PASI improvement could be increased by adding diet restriction protocols, mainly the low-calorie diet, to the course of pharmacological therapies [24]. In obese patients with CPP, despite non-significant improvements in STG, besides the significant decrease in patients’ weight and WC, adding an 8-week low-calorie diet (≤ 1000 kcal/day) to biological therapies (infliximab, etanercept, adalimumab, and ustekinumab) achieved greater improvement in PASI than biological therapies alone [25].

Regarding ED improvement, adherence to weight-loss programs including diet restriction and/or increased physical activity augments a strong penile erection in ED sufferers, likely via improving mood and self-esteem, increasing serum testosterone levels [26], regulating the disturbances of metabolic profile (proinflammatory markers, insulin resistance, and dyslipidemia), decreasing the mass of visceral adipose tissue, improving vascular functions (improving endothelial dysfunction, increased NO production, and increased local blood supply to the penis) [27], increasing relaxation of smooth muscular tissues of the penis, and inducing neuro-biochemical/hormonal changes involved in the process of penile erection [12].

Esposito et al. [28] supported our results because their results were compatible with our results, especially after their random assignment of 110 ED men to an intervention group (*n* = 55 obese men, the role of the authors in this group was to guide men how to lose 10% of their weight over one year through a detailed prescription of restricting diet calories and how to increase exercise levels) or control group (*n* = 55 obese men, the role of the authors in this group was to guide men how to lose 10% of their weight over one year through a piece of general non-detailed information about diet restriction or increasing the daily exercise levels). Esposito et al. [28] showed that the intervention group produced higher significant improvement in the tested outcomes (weight, BMI, SHDL, STG, blood pressure, IIEFQ-5, and glucose) than the control group.

Esposito et al. [29] supported our results because their results were compatible with our results, especially after their random assignment of 209 obese men to a 104-men intervention group (34% of men had IIEFQ-5 > 21, the role of the authors in this group was to guide men to how to lose their weight over two years through a detailed prescription on how to restrict calories of diet and how to increase exercise levels) or 105-men control group (36% of men had IIEFQ-5 > 21, the role of the authors in this group was to guide men to lose their weight over two years through a piece of general non-detailed information about diet restriction or increasing the daily exercise levels). Besides the trend of improvement in weight, BMI, SHDL, STG, blood pressure, and blood glucose in favor of the intervention group, after ending the 2-year period, Esposito et al. [29] showed that the percentage of men in the intervention group who had normal IIEFQ-5 > 21 increased to 56% while the percentage of men in the control group who had normal IIEFQ-5 > 21 increased to 38%.

The response of IIEFQ-5 to an 8-week interval exercise in hypertensive men with ED was compatible with our results because IIEFQ-5 significantly increased after the interval exercise program [30]. Again, besides the significant improvement in BMI, insulin resistance, WC, BP, SHDL, and STG, an 8-week elliptical exercise increased the efficacy of ED pharmacotherapies in obese men with mild and moderate ED [12].

Except for WC and diastolic BP, comparing the post results of obese men with ED who followed a Mediterranean diet protocol with the post results of obese men with ED who did not follow any calorie restriction diet showed significant improvements in systolic BP, SHDL, STG, blood glucose, and IIEFQ-5 in favor of Mediterranean diet men [31].

The results of the random assignment of overweight and obese diabetic men with ED to a group of an intensive lifestyle-modification program (diet restriction with exercise) or a group of diabetes education/support program were compatible with our results due to the trend of improvement in tested outcomes (weight, glycosylated hemoglobin, BP, IIEFQ-5, and SHDL) was in favor of the group of the intensive lifestyle modification program [32].

Opposite to us, despite augmenting the high efficacy of ED pharmacotherapies in the treatment of ED in MeTS men with ED, adding exercise to ED pharmacotherapies did not augment significant improvements in BP, WC, SHDL, STG, and blood glucose due to including a small number (*n* = 10) of MeTS elderly with ED [33].

## Limitations

Comparing the effect of different types of calorie restriction diets on psoriasis severity and psoriasis-associated ED and MeTS is the main limitation of this study. The two authors invite researchers of CPP to cover this limitation in future studies.

## Conclusion

The 12-week lifestyle modification program as a tool for weight loss in obese patients with CPP is a good therapeutic method to improve psoriasis severity and psoriasis-associated ED and MeTS.

## Data Availability

Data (age, WC, BMI, IIEFQ-5, PASI, BP, STG, SFBG, and SHDL) will be available on request.
